# Impact of Latent Tuberculosis Infection on Ovarian Reserve and In Vitro Fertilization or Intracytoplasmic Sperm Injection Outcomes: A Retrospective Cohort Study with Propensity Score Matching

**DOI:** 10.7150/ijms.114851

**Published:** 2025-10-20

**Authors:** Yifan Chu, Jialiang Zhang, Luyao Wang, Jiaxin Xie, Jiayun Chen, Miao Yan, Xinyao Hu, Bo Zhang, Jing Yue

**Affiliations:** 1 Reproductive Medicine and Genetics Centre, Tongji Hospital, Tongji Medical College, Huazhong University of Science and Technology, Wuhan, People's Republic of China.; 2 Department of Obstetrics and Gynecology, Tongji Hospital, Tongji Medical College, Huazhong University of Science and Technology, Wuhan, People's Republic of China.; 3 Hubei Clinical Research Center for Reproductive Medicine. Shiyan, People's Republic of China.; 4 Shiyan Key Laboratory of Reproduction and Genetics (Renmin Hospital, Hubei University of Medicine). Shiyan, People's Republic of China.; 5 School of Mechanical Science and Engineering, Huazhong University of Science and Technology. Wuhan, People's Republic of China.; 6 National Clinical Research Center for Obstetrics and Gynecology, Cancer Biology Research Center (Key Laboratory of the Ministry of Education), Tongji Hospital, Tongji Medical College, Huazhong University of Science and Technology. Wuhan, People's Republic of China.

**Keywords:** latent tuberculosis infection, ovarian reserve, pregnancy outcomes, in vitro fertilization, intracytoplasmic sperm injection, propensity score matching

## Abstract

**Background**: Tuberculosis is a communicable disease that is a major cause of ill health and one of the leading causes of death worldwide. Latent tuberculosis infection (LTBI) widely exists in people all over the world, especially in patients with unexplained infertility, and the relationship between latent tuberculosis infection and ovarian reserve, as well as pregnancy outcomes of in vitro fertilization/intracytoplasmic sperm injection (IVF/ICSI), remains poorly understood.

**Methods:** A single-center, retrospective cohort study was conducted at the Reproductive Medicine and Genetics Centre, Tongji Hospital, Tongji Medical College, Huazhong University of Science and Technology, between January 2018 and December 2020. The study aimed to investigate whether LTBI affects ovarian reserve and pregnancy outcomes in infertile women undergoing assisted reproductive technology. The primary outcomes were ovarian reserve and cumulative live birth rate per IVF/ICSI cycle, while secondary outcomes included pregnancy outcomes and maternal and neonatal complications.

**Results:** A total of 11523 assisted reproductive technology cycles were ultimately included in the comparison of ovarian reserves, and 9141 IVF/ICSI cycles were ultimately included in the comparison of clinical outcomes between the LTBI and control groups. The data revealed that women with LTBI had significantly lower anti-Müllerian hormone (4.61 ± 3.99 ng/mL vs. 4.88 ± 4.22 ng/mL, P=0.035, β=-0.23, 95% CI -0.43 to -0.04) and antral follicle counts [11.00 (8.00, 17.00) vs. 12.00 (8.00, 19.00), P=0.048, β=-0.26, 95% CI -0.53 to -0.01]. The conservative and optimistic cumulative live birth rates (61.42% vs. 61.94%, adjusted OR: 0.95, 95% CI: 0.82-1.10; 72.65% vs. 73.25%, adjusted OR: 0.94, 95% CI: 0.78-1.14), the live birth rates after fresh embryo transfer (39.28% vs. 40.83%, adjusted OR: 0.97, 95% CI: 0.82-1.14) and other secondary outcomes in the LTBI group were comparable to those in the control group after excluding factors such as age, ovarian reserve, and the number of oocytes retrieved.

**Conclusions:** LTBI may affect the ovarian reserve but not directly affect the pregnancy outcomes of IVF/ICSI in infertile women.

## Introduction

Tuberculosis (TB), an infectious disease caused by *Mycobacterium tuberculosis* (MTB), constitutes a persistent global health threat and remains one of the leading causes of mortality worldwide. The estimated global TB incidence reached 10.8 million cases in 2023, reflecting a marginal increase from 10.7 million cases in 2022. With a global incidence rate of approximately 134 cases per 100,000 people, TB imposes a substantial burden of disease. Critically, TB claimed approximately 1.25 million lives globally in 2023 alone. This mortality burden establishes TB as the foremost single infectious cause of death worldwide, exceeding even human immunodeficiency virus (HIV)/acquired immune deficiency syndrome (AIDS) fatalities by nearly twofold 1.

Latent tuberculosis infection (LTBI) is an asymptomatic chronic condition characterized by a sustained immune response to MTB antigens in the absence of clinical signs of active TB 2, 3. Epidemiological studies indicate considerable variations in the prevalence of LTBI across different populations. In China, a multicenter prospective cohort study estimated a national LTBI prevalence of 18.8% 4. Higher rates have been observed among high-risk groups: household contacts of TB patients show LTBI prevalence ranging from 32% to 48%, while healthcare workers in high-incidence settings exhibit even broader variation, between 15% and 70% 5-7. Notably, individuals with active TB may transition to LTBI after receiving anti-TB treatment. Conversely, approximately 5% to 10% of LTBI cases progress to active TB over the course of a lifetime, contributing to new sources of TB infection 8, 9.

An estimated 5%-13% of reproductive-age women harbor LTBI 10. In high-TB-burden regions such as China and India, clinical screening indicates that approximately 9%-27.1% of infertile patients are affected by LTBI, underscoring significant clinical risks 11, 12. Previous studies have demonstrated that patients with genital TB associates with diminished ovarian reserve (OR), fewer oocytes, poorer embryo quality, and impaired subendometrial blood flow 13-16. However, the relationships between LTBI and OR, as well as assisted reproductive technology (ART) pregnancy outcomes, remain inadequately characterized. Our prior cohort study revealed that LTBI had no significant impact on OR or pregnancy outcomes in infertile women undergoing intrauterine insemination 12. By contrast, two retrospective studies from China have suggested that patients with LTBI exhibit thinner endometrium; lower implantation rates, clinical pregnancy rates, and live birth rates; and elevated miscarriage rates than control patients do following *in vitro* fertilization/intracytoplasmic sperm injection (IVF/ICSI) with fresh embryo transfers 17, 18. These studies were limited by small sample sizes, varying LTBI diagnostic methods, and a focus solely on fresh embryo transfer cycles. Consequently, this large- scale retrospective cohort study aims to elucidate the effects of LTBI on OR and IVF/ICSI pregnancy outcomes in infertile women, providing valuable insights for clinical practice.

## Methods

### Study population and participants

Patients who were receiving their first ART at the Reproductive Medicine and Genetics Center, Tongji Hospital, Tongji Medical College, Huazhong University of Science and Technology, between January 2018 and December 2020 were enrolled for comparison of ORs between the LTBI and control groups. Before initiating ART, all patients underwent venous blood sampling for interferon-gamma release assay (IGRA), utilizing either the T-SPOT.TB test (Oxford Immunotec Ltd.) or the QuantiFERON-TB Gold assay (Qiagen, Germany). IGRA positive patients were diagnosed as LTBI after excluding active TB by negative erythrocyte sedimentation rate and C-reactive protein, while IGRA negative patients were classified as the control group. The exclusion criteria were as follows: 1) non- first ART cycles; 2) incomplete or missing clinical data; 3) indeterminate IGRA results; 4) the presence of ovarian cysts, tumors, or a history of ovarian surgery; 5) active TB; and 6) chromosomal abnormalities.

Patients who underwent their first IVF/ICSI treatment at the Reproductive Medicine and Genetics Center, Tongji Hospital, Tongji Medical College, Huazhong University of Science and Technology, between January 2018 and December 2020 were included in the comparison of IVF/ICSI outcomes between the LTBI and control groups. The participants were divided into LTBI and control groups on the basis of the IGRA results. The exclusion criteria were as follows: 1) non-first ART cycles; 2) incomplete or missing clinical data; 3) indeterminate IGRA results; 4) active TB; 5) cycle cancellation; 6) preimplantation genetic testing (PGT); 7) use of testicular sperm aspiration (TESA)/percutaneous epididymal sperm aspiration (PESA)/microdissection testicular sperm extraction (mTESE); 8) receipt of donor oocytes for IVF/ICSI; 9) use of frozen sperm or oocytes in the cycle; and 10) no available embryos for transfer. Each IVF/ICSI cycle was followed for a period of 2 years. Data were extracted from the electronic medical records database.

### IVF/ICSI Protocol

Controlled ovarian hyperstimulation (COH) protocols primarily include gonadotropin-releasing hormone (GnRH) agonists, GnRH antagonists, progestin-primed ovarian stimulation (PPOS), and other protocols, such as microstimulation and natural cycles. In addition to natural cycles, the initial gonadotropin (Gn) dose typically ranges from 112.5 to 300 IU/day, which is determined on the basis of the patient's OR, body mass index (BMI), COH protocol, and other factors. During COH, transvaginal ultrasonography (TVS) was performed every 2-4 days to monitor follicular development. The protocols for follicular monitoring, oocyte retrieval, embryo culture and transfer, and luteal support were consistent with those used in our previous study 19.

### Primary and secondary outcomes

The primary outcome measures used in this study included anti-Müllerian hormone (AMH) levels, the antral follicle count (AFC), basal follicle-stimulating hormone (bFSH) levels, fresh-cycle live birth rates, and both conservative and optimistic cumulative live birth rates (CLBRs) per IVF/ICSI cycle. The secondary outcomes included the biochemical pregnancy rate, clinical pregnancy rate, miscarriage rate, ectopic pregnancy rate, multiple pregnancy rate, preterm birth rate, and rates of maternal and neonatal complications during the fresh cycle, as well as conservative and optimistic cumulative pregnancy rates (CPRs) per IVF/ICSI cycle. Venous blood was collected from the enrolled patients on the Day 2-4 of the menstrual cycle to detect the AMH and bFSH levels (Kaeser 6600 immunoassay analyzer, Kangrun Biotech, China). Besides, our experienced ultrasound physicians used the color Doppler ultrasound diagnostic system (DD60/DF37, DIT, China) to record the number of antral follicles with diameters ranging from 2-9 mm in both ovaries. Biochemical pregnancy was defined as a serum β-hCG concentration of ≥10 IU/mL measured 12-14 days after embryo transfer. Clinical pregnancy was defined as the presence of an intrauterine pregnancy sac visible via TVS at 28 days after embryo transfer, irrespective of the presence or absence of embryonic cardiac activity. Miscarriage was defined as pregnancy loss occurring before 28 weeks of gestation. Live birth was defined as the delivery of at least one live infant after 28 weeks of gestation. Preterm birth was defined as the delivery of at least one live infant between 28 and 36^+6^ weeks of gestation. Multiple pregnancies were defined as the presence of two or more gestational sacs confirmed by TVS 20. For cumulative outcomes, the optimistic CPR or CLBR assumed that patients with remaining frozen embryos that had not been transferred or who had not undergone further treatment cycles had the same likelihood of achieving a clinical pregnancy or live birth as those who had undergone embryo transfer. In contrast, conservative CPR or CLBR assumes that no clinical pregnancies or live births occurred in the untransferred cycles 21, 22. Maternal and neonatal complications included hypertensive disorders of pregnancy, gestational diabetes mellitus, fetal distress, placenta previa, placental abruption, premature rupture of membranes, fetal growth restriction, intrahepatic cholestasis of pregnancy, postpartum hemorrhage, neonatal low birth weight, macrosomia, neonatal pneumonia, pathological jaundice in the newborn, and congenital birth defects.

### Statistical analysis

R software (version 4.3.0) was used for propensity score matching (PSM) and subsequent statistical analyses in both the LTBI group and the control group. Standardized mean differences (SMDs) exceeding 0.1 were used as independent variables in the PSM model, with a caliper width of 0.1 applied at matching ratios of 1:2 for comparisons of OR and 1:3 for IVF/ICSI pregnancy outcomes. Continuous variables are presented as the mean ± SD or median (25th percentile, 75th percentile). Normality tests were conducted to determine whether differences between the two groups were statistically significant via either Student's t test or the nonparametric Wilcoxon rank-sum test, as appropriate. Categorical variables are expressed as percentages. Chi-square tests or Fisher's exact tests were employed to assess whether differences between the two groups were statistically significant. Multivariate linear regression was applied to analyze the factors influencing the OR. Multivariate logistic regression was used to examine factors affecting conservative and optimistic CPR/CLBR, as well as clinical pregnancy, live birth, and miscarriage outcomes in the fresh cycle. Two-tailed P values <0.05 were considered statistically significant.

## Results

### Analysis 1: Comparison of ORs between the LTBI and control groups

**Figure 1:**
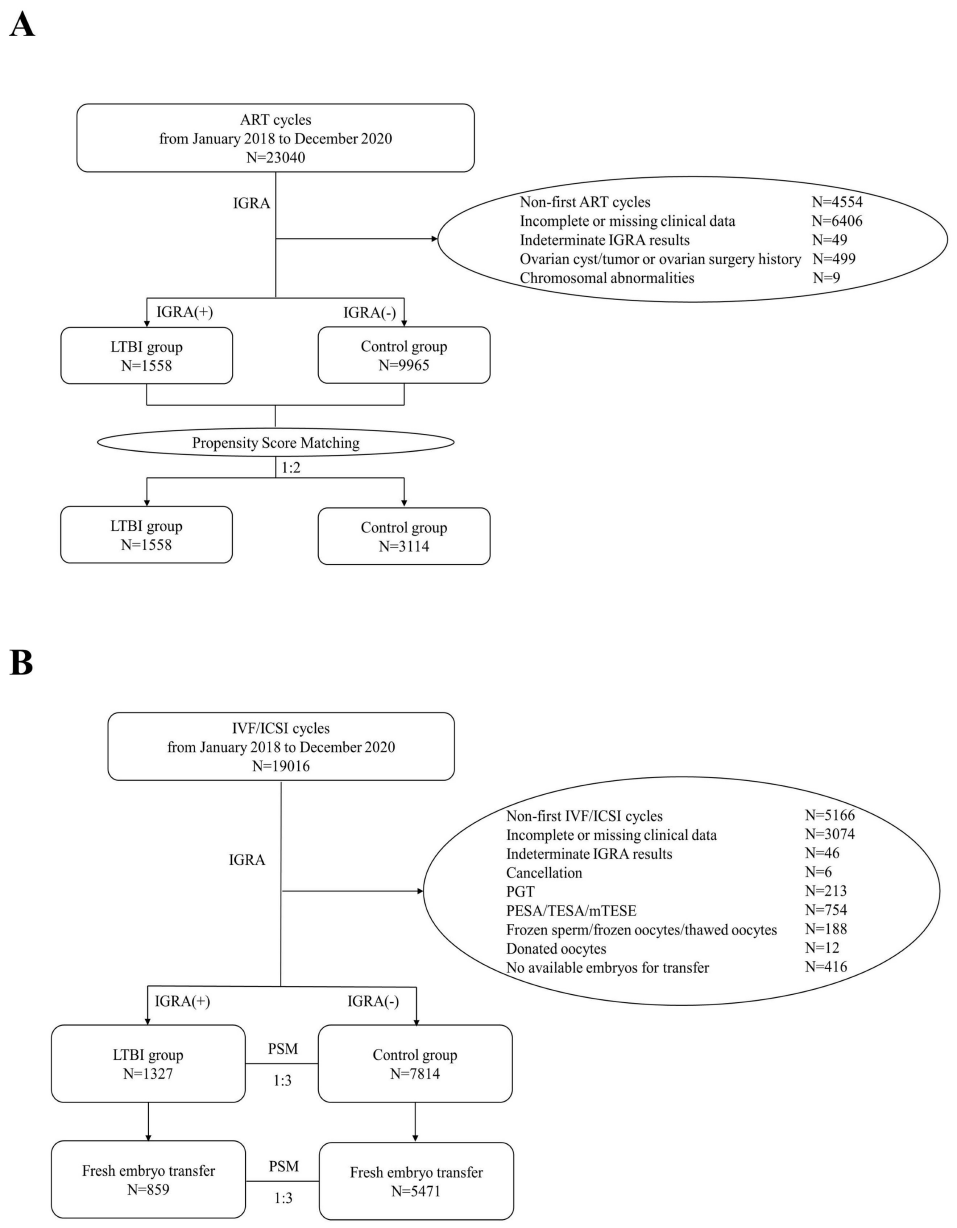
Flowchart of participants included in this study. A) Flowchart of the selection of cases for comparing OR between LTBI and control groups. B) Flowchart for the selection of cases for comparing IVF/ICSI pregnancy outcomes between LTBI and control groups.

In this study, a total of 23,040 ART cycles conducted from January 2018 to December 2020 were retrospectively reviewed. After excluding 4,554 cycles of non-first ART attempts, 6,406 cycles with incomplete information, 49 cycles with indeterminate IGRA results, 499 cycles involving patients with ovarian cysts/tumors or a history of previous ovarian surgery, and 9 cycles involving patients with chromosomal abnormalities, a total of 11,523 patients were ultimately included in the analysis. Among these, 1,558 patients were assigned to the LTBI group, and 9,965 patients composed the control group. PSM was performed at a ratio of 1:2 to balance the baseline characteristics between the groups (**Figure 1A**).

Compared with controls, LTBI group patients were significantly older (31.93 ± 4.70 years vs. 30.79 ± 4.34 years, P<.001) and exhibited higher BMI (22.11 ± 3.02 kg/m² vs. 21.90 ± 3.06 kg/m², P=0.011). Additionally, the LTBI group demonstrated increased rates of secondary infertility (30.87% vs. 27.08%, P=0.002) and higher prevalence of pelvic/tubal factors (52.31% vs. 41.60%, P<.001) and uterine factors (20.54% vs. 18.07%, P=0.020). Conversely, lower frequencies were observed in the LTBI group for endometriosis associated infertility (5.20% vs. 6.57%, P=0.039), male factor infertility (29.65% vs. 36.78%, P<.001), and unexplained infertility (7.70% vs. 10.85%, P<.001). Additionally, IVF/ICSI utilization was significantly more prevalent among LTBI patients (88.51% vs. 82.17%, P<.001). Age, BMI, ART method, type of infertility, pelvic/tubal factor associated infertility, endometriosis associated infertility, male factors associated infertility, uterine factors associated infertility, and unexplained infertility were included in the PSM model. After PSM, there were no statistically significant differences in the baseline characteristics between the two groups, except for the etiological component related to endometriosis, as detailed in **Table 1**.

After PSM, the ORs were compared between the two groups, as detailed in **Table 2**. The levels of AMH and AFC were significantly lower in the LTBI group than in the control group [AMH: 4.61 ± 3.99 ng/mL vs. 4.88 ± 4.22 ng/mL, P=0.035; AFC: 11.00 (8.00, 17.00) vs. 12.00 (8.00, 19.00), P=0.048]. However, there was no significant difference in bFSH levels between the two groups (7.92 ± 2.91 mIU/mL vs. 7.80 ± 3.08 mIU/mL, P=0.219). Multivariate linear regression analysis revealed that LTBI was an independent risk factor for decreases in both AMH and the AFC (P=0.019, β=-0.23, 95% CI -0.43 to -0.04; P=0.049, β=-0.26, 95% CI -0.53 to -0.01), as shown in **Table 3**.

Given the substantial heterogeneity in the clinical manifestations of polycystic ovary syndrome (PCOS) and its significant influence as a confounding factor on AMH and AFC, we further analyzed 7,867 non-PCOS-related infertile women in the control group and 1,261 non-PCOS-related infertile women in the LTBI group. As shown in **Tables S1-S2**, after PSM, the comparison of ORs between the two groups yielded results consistent with our previous findings. Furthermore, the results indicated that both the AMH and AFC levels were significantly lower in the LTBI group than in the control group (AMH: 3.41 ± 2.59 ng/mL vs. 3.78 ± 2.89 ng/mL, P<.001; AFC: 10.84 ± 5.20 vs. 11.39 ± 5.18, P<.001).

### Analysis 2: Comparison of IVF/ICSI outcomes between the LTBI and control groups

In this study, a total of 19,016 IVF/ICSI cycles conducted from January 2018 to December 2020 were retrospectively reviewed. After excluding 5,166 cycles of non-first IVF/ICSI attempts; 3,074 cycles with incomplete information; 46 cycles with indeterminate IGRA results; 6 cycles that were canceled; 213 cycles involving PGT; 754 cycles involving PESA/TESA/mTESE; 188 cycles involving frozen sperm, frozen oocytes, or thawed oocytes; 12 cycles involving donated oocytes; and 416 cycles with no available embryos, a total of 9,141 IVF/ICSI cycles were ultimately included in the analysis. Among these, 1,327 cycles were assigned to the LTBI group (including 859 cycles with fresh embryo transfer), and 7,814 cycles composed the control group (including 5,471 cycles with fresh embryo transfer). PSM was performed at a ratio of 1:3 to balance the baseline characteristics between the groups (**Figure 1B**).

The baseline characteristics of the two groups are summarized in **Table 4**. Before PSM, compared with those in the control group, patients in the LTBI group were significantly older (32.12 ± 4.63 years vs. 31.05 ± 4.32 years, P<.001) and had higher bFSH levels (7.90 ± 2.68 mIU/mL vs. 7.66 ± 2.58 mIU/mL, P=0.002). Additionally, the LTBI group presented lower levels of AMH [3.35 (1.69, 5.87) ng/mL vs. 3.59 (1.92, 6.40) ng/mL, P<.001] and AFC [12.00 (8.00, 18.00) vs. 12.00 (8.00, 19.00), P<.001]. Furthermore, the LTBI group had higher rates of pelvic/tubal factors (60.29% vs. 49.74%, P<.001) and uterine factors (23.13% vs. 20.67%, P=0.041) but lower rates of endometriosis-related infertility (6.48% vs. 8.20%, P=0.032), male factor infertility (23.89% vs. 29.59%, P<.001), and unexplained infertility (6.25% vs. 9.12%, P<.001). In COH, the proportion of patients receiving the GnRH agonist protocol was lower in the LTBI group (57.35% vs. 61.10%, P=0.029). Additionally, the proportion of ICSI cycles was significantly lower in the LTBI group (24.72% vs. 29.59%, P<.001). Age, bFSH, AFC, AMH, pelvic/tubal factor associated infertility, endometriosis associated infertility, male factors associated infertility, uterine factors associated infertility, unexplained infertility, COH protocol and fertilization methods were included in the PSM model. After PSM, the rate of endometriosis associated infertility in the LTBI group was significantly lower than that in the control group (6.48% vs. 8.58%, P=0.015), and no statistically significant differences were observed in other baseline characteristics between the two groups (P>0.05).

As shown in **Table 5**, following COH, there were no significant differences between the LTBI group and the control group in terms of Gn duration, Gn dosage, the number of follicles >14 mm on the hCG day, the number of oocytes retrieved, the number of metaphase II (MII) oocytes, or the number of double pronucleus (2PN) embryos (P>0.05). Additionally, no statistically significant differences were observed in blastocyst formation rates or the number of available embryos between the two groups. The conservative and optimistic CLBRs (61.42% vs. 61.94%, P=0.733; 72.65% vs. 73.25%, P=0.665) and CPRs (70.16% vs. 70.42%, P=0.857; 76.26% vs. 77.18%, P=0.494) in the LTBI group were also comparable to those in the control group. Multivariate logistic regression analysis of factors influencing the CPR and CLBR revealed that LTBI was not an independent risk factor for either conservative or optimistic CPR or the CLBR when the OR was equivalent, as detailed in **Table 6**.

To evaluate the direct impact of LTBI on pregnancy outcomes following fresh embryo transfer, we further analyzed 859 patients in the LTBI group and 5471 patients in the control group who underwent fresh embryo transfer. As shown in **Table S3**, after PSM, the proportion of patients with endometriosis-related infertility in the LTBI group was significantly lower than that in the control group (5.94% vs. 8.49%, P=0.017), and no statistically significant differences in other baseline characteristics were observed between the two groups (P>0.05).

As presented in **Table S4 and Table 7**, the COH outcomes and pregnancy outcomes were comparable between the two groups, with no statistically significant differences observed in the live birth rate after fresh embryo transfer (39.28% vs. 40.83%, P=0.424). Similarly, other pregnancy outcomes and the incidence of maternal and infant complications did not differ significantly between the groups (P>0.05). Furthermore, multivariate logistic regression analysis revealed that when the OR was equivalent, LTBI was not identified as an independent risk factor for clinical pregnancy, live birth, or miscarriage following fresh embryo transfer, as detailed in **Table 8**.

## Discussion

The effects of genital TB on female reproductive health are well documented 13-16. Nevertheless, the association between the high prevalence of LTBI and OR in women, as well as IVF/ICSI pregnancy outcomes, remains a topic of debate. Li *et al*. reported that LTBI patients had a thinner endometrium than control patients did and experienced significantly lower implantation rates, clinical pregnancy rates, and live birth rates following IVF/ICSI with fresh embryo transfers, while also having a higher abortion rate 17. Similarly, Jia *et al*. reported that clinical pregnancy rates following IVF/ICSI with fresh embryo transfers were significantly lower in the LTBI group than in the control group, although no statistically significant differences were found in live birth rates or abortion rates 18. However, our study revealed that LTBI may affect the OR but not directly affect the pregnancy outcomes of IVF/ICSI. It is particularly noteworthy that the majority of the aforementioned studies employed conventional diagnostic methods such as the tuberculin skin test or chest X-ray for LTBI screening. These techniques are limited by their low specificity. False-positive results may occur in individuals with a history of bacille Calmette-Guérin vaccination or exposure to *Mycobacterium bovis* and non-tuberculous mycobacteria. Conversely, false-negative outcomes can arise under conditions such as recent MTB infection, co-existing bacterial or viral infections, malnutrition, hypoproteinemia, immunosuppressive states, and advanced age 23. With advancements in immunology, molecular biology, and other fields, the IGRA has been increasingly applied in clinical practice. This diagnostic tool detects interferon-gamma produced by sensitized T lymphocytes in whole blood or isolated from whole blood after re-exposure to MTB-specific antigens, enabling the identification of TB infection 24. IGRA offers several advantages, including high specificity, standardized test procedures, low error rates, and high detection efficiency. These attributes have led to its recommendation as a screening method by the World Health Organization and multiple clinical guidelines in the field of TB screening 25-28. Notably, previous research has demonstrated that patients diagnosed with genital TB exhibit comparable IVF success rates to those without genital TB, assuming normal uterine volume and morphology 29. Among patients with LTBI in our center, only 0.9% were diagnosed with genital TB (unpublished data). These patients were diagnosed with genital TB via hysteroscopy and endometrial pathological examination, which was initially indicated due to abnormal TVS findings such as intrauterine occupying lesions, intrauterine adhesions, or abnormal uterine bleeding, and recurrent implantation failure. Following standardized anti-TB treatment, their clinical outcomes demonstrated significant improvement. Considering the extremely low prevalence of genital TB among LTBI patients and the observation that women eligible for embryo transfer typically exhibit optimal endometrial conditions, we propose that LTBI does not have a direct effect on the pregnancy outcomes of IVF/ICSI. Finally, it is noteworthy that variability among commercial assay kits and laboratory protocols may influence the diagnostic accuracy for LTBI, which, albeit with a low probability, could potentially lead to biased assessments of its association with adverse outcomes.

Numerous factors influence the outcomes of IVF/ICSI cycles. These include patient age, COH protocols, endometrial thickness on the hCG day, and the number and quality of transferred embryos, all of which are associated with the pregnancy outcomes of fresh embryo transfer cycles. Additionally, factors such as age, the number of oocytes retrieved, embryo quality, and the number of available embryos are correlated with the CLBR per IVF/ICSI cycle 30-33. This study revealed that LTBI may adversely affect women's OR, potentially decreasing the quantity and quality of oocytes and embryos available for IVF/ICSI. Mogombedze *et al*. comprehensively reviewed the mechanisms underlying the metabolic plasticity induced by LTBI and the adaptive strategies employed by MTB in response to host immune dynamics, leading to the inference that the ovarian microenvironment, characterized by its lipid-rich conditions and nitric oxide production, may provide a favorable niche for TB infection 34. Notably, this immune-mediated tissue damage associated with LTBI can result in localized ovarian fibrosis and vascular compromise, potentially accelerating pathological ovarian aging. These findings underscore the importance of vigilant monitoring and assessment of ORs in women with LTBI. Furthermore, these findings emphasize the need for women with LTBI to adopt a healthy lifestyle, plan family timelines judiciously, and address ovarian aging-related endocrine and infertility issues promptly to preserve reproductive health. Furthermore, the findings of this study may alleviate concerns among LTBI patients with normal ORs regarding adverse IVF/ICSI pregnancy outcomes, as stress or depression are also significant factors influencing IVF/ICSI success 35.

In developing countries such as India and China, the prevalence of LTBI has been steadily increasing. According to the data from this cohort, the prevalence of LTBI among infertile women undergoing ART at our center was observed to be 13.5%. Although this figure is lower than some previously reported estimates in the literature, screening and management of LTBI remain critically important due to its potential progression to active TB during pregnancy, resulting in adverse pregnancy outcomes 4, 36, 37. Several factors, such as hyperphysiological hormone levels during COH, the potential reactivation of old pelvic lesions during oocyte retrieval, immune function changes caused by luteal support drugs, and the inherent decline in immune function during pregnancy, not only increase the susceptibility of pregnant women to new TB infections but also may reactivate LTBI 38, 39. For patients diagnosed with active TB before pregnancy, clinicians should advise standard anti-TB treatment and recommend deferring ART to minimize risks. For those with LTBI identified before ART, clinicians must inform them of potential adverse outcomes that may arise during pregnancy. When symptoms such as unexplained fever, cough, sputum, or vaginal bleeding emerge during pregnancy and conventional treatments prove ineffective, healthcare providers should promptly consider the possibility of active TB complicating the pregnancy. Timely diagnosis and appropriate treatment are essential, and in some cases, pregnancy termination may be necessary to safeguard a woman's health.

To our knowledge, this is the largest clinical study to date investigating the effects of LTBI on OR and IVF/ICSI outcomes. Compared with previous studies, we conducted a more in-depth analysis of the potential impact of LTBI on IVF/ICSI pregnancy outcomes through the assessment of the CLBR and CPR. While our study contributes valuable insights, it is not without its limitations. First, as a single-center, retrospective cohort study, it is subject to methodological constraints, including a limited level of evidence and sample size. Second, this study was confined to infertile individuals undergoing ART, thereby limiting the generalizability of the findings to the broader population. Additionally, the impact of LTBI on reproductive function within the general population remains uncertain. Moreover, the assessment of OR was primarily based on a single cross-sectional analysis conducted at the time of initial diagnosis, and a long-term follow-up study involving a large cohort was not feasible. Furthermore, owing to the retrospective nature of this study, it was challenging to conclusively exclude the potential influence of confounding variables, including genetic, environmental, psychological, and lifestyle factors, on OR. Future research should involve large-scale, prospective cohort studies and a series of basic experimental investigations to further elucidate the effects and potential mechanisms of LTBI on OR and reproductive outcomes.

## Conclusion

LTBI may affect the ovarian reserve but not directly affect the pregnancy outcomes of IVF/ICSI in infertile women. We strongly recommend that infertile women undergo routine TB screening before receiving ART, and patients with LTBI should undergo an assessment of ovarian reserve to develop a rational family plan. For women with a normal ovarian reserve, clinicians should endeavor to alleviate their anxiety and other negative emotions to prevent potential adverse effects on IVF/ICSI pregnancy outcomes.

## Supplementary Material

Supplementary tables.

## Figures and Tables

**Table 1:** Baseline characteristics of the women included in Analysis 1^*^

	Before PSM					After PSM				
	Control group (n = 9965)	LTBI group (n = 1558)	Statistics	*P*	SMD	Control group (n = 3114)	LTBI group (n = 1558)	Statistics	*P*	SMD
Age (years)	30.79 ± 4.34	31.93 ± 4.70	t=-8.997	<.001^†^	0.243	31.91 ± 4.66	31.93 ± 4.70	t=-0.113	0.910	0.003
BMI (kg/m^2^)	21.90 ± 3.06	22.11 ± 3.02	t=-2.556	0.011^†^	0.070	21.98 ± 2.98	22.11 ± 3.02	t=-1.395	0.163	0.043
Duration of infertility (years)	3.00 (2.00, 4.00)	3.00 (2.00, 4.00)	Z=-1.264	0.206	0.084	3.00 (1.50, 4.00)	3.00 (2.00, 4.00)	Z=-1.651	0.099	0.071
ART method, n (%)			χ²=38.468	<.001^†^	0.199			χ²=0.000	0.994	0.000
Artificial Insemination	1777 (17.83)	179 (11.49)				358 (11.50)	179 (11.49)			
IVF/ICSI	8188 (82.17)	1379 (88.51)				2756 (88.50)	1379 (88.51)			
Type of infertility, n (%)			χ²=9.676	0.002^†^	0.082			χ²=0.776	0.378	0.028
Primary infertility	7266 (72.92)	1077 (69.13)				2113 (67.85)	1077 (69.13)			
Secondary infertility	2699 (27.08)	481 (30.87)				1001 (32.15)	481 (30.87)			
Cause of Infertility, n (%)										
Ovulation Dysfunction (except PCOS)	1201 (12.05)	206 (13.22)	χ²=1.720	0.190	0.035	416 (13.36)	206 (13.22)	χ²=0.017	0.897	0.004
PCOS	2098 (21.05)	297 (19.06)	χ²=3.243	0.072	0.051	623 (20.01)	297 (19.06)	χ²=0.585	0.445	0.024
Pelvic/Tubal Factor	4145 (41.60)	815 (52.31)	χ²=63.098	<.001^†^	0.215	1630 (52.34)	815 (52.31)	χ²=0.000	0.983	0.001
Endometriosis	655 (6.57)	81 (5.20)	χ²=4.254	0.039^†^	0.062	224 (7.19)	81 (5.20)	χ²=6.769	0.009^†^	0.090
Male Factor	3665 (36.78)	462 (29.65)	χ²=29.757	<.001^†^	0.156	920 (29.54)	462 (29.65)	χ²=0.006	0.938	0.002
Uterine Factor	1801 (18.07)	320 (20.54)	χ²=5.455	0.020 ^†^	0.061	690 (22.16)	320 (20.54)	χ²=1.606	0.205	0.040
Unexplained	1081 (10.85)	120 (7.70)	χ²=14.281	<.001^†^	0.118	241 (7.74)	120 (7.70)	χ²=0.002	0.964	0.001

* Values are presented as the mean±SD, median (25th percentile, 75th percentile) or proportion (%). ^†^*P*<0.05 Abbreviations: PSM, propensity score matching; LTBI, latent tuberculosis infection; SMD, standardized mean difference; BMI, body mass index; ART, assisted reproductive technology; IVF, *in vitro* fertilization; ICSI, intracytoplasmic sperm injection; PCOS, polycystic ovary syndrome.

**Table 2:** Comparisons of AMH, bFSH and AFC between the LTBI group and the control group^*^

	Control group (n = 3114)	LTBI group (n = 1558)	Statistics	*P*
AMH (ng/mL)	4.88 ± 4.22	4.61 ± 3.99	t=2.11	0.035^†^
bFSH (mIU/mL)	7.80 ± 3.08	7.92 ± 2.91	t=-1.23	0.219
AFC	12.00 (8.00, 19.00)	11.00 (8.00, 17.00)	Z=0.47	0.048^†^

^*^ Values are presented as the mean±SD or median (25th percentile, 75th percentile). ^†^ *P*<0.05 Abbreviations: LTBI, latent tuberculosis infection; AMH, anti-Müllerian hormone; bFSH, basal follicle stimulating hormone; AFC, antral follicle count.

**Table 3:** Multivariate linear regression analysis of the related factors affecting OR

Variate	AMH		AFC		bFSH	
	*P*	β (95%CI)	*P*	β (95%CI)	*P*	β (95%CI)
Age	<.001	-0.13 (-0.15 ~ -0.11)	<.001	-0.30 (-0.33 ~ -0.27)	<.001	0.09 (0.07 ~ 0.11)
LTBI	0.019	-0.23 (-0.43 ~ -0.04)	0.049	-0.26 (-0.53 ~ -0.01)	0.190	0.12 (-0.06 ~ 0.29)
Ovulation Dysfunction (except PCOS)	<.001	-1.82 (-2.10 ~ -1.55)	<.001	-4.97 (-5.37 ~ -4.57)	<.001	1.91 (1.66 ~ 2.16)
PCOS	<.001	5.52 (5.28 ~ 5.76)	<.001	11.45 (11.10 ~ 11.79)	<.001	-0.92 (-1.13 ~ -0.70)
Endometriosis	0.001	-0.62 (-0.99 ~ -0.25)	<.001	-1.81 (-2.34 ~ -1.27)	0.016	0.41 (0.08 ~ 0.74)

Abbreviations: OR, ovarian reserve; AMH, anti-Müllerian hormone; AFC, antral follicle count; bFSH, basal follicle stimulating hormone; LTBI, latent tuberculosis infection; PCOS, polycystic ovary syndrome.

**Table 4:** Baseline characteristics of the women included in Analysis 2^*^

	Before PSM					After PSM				
	Control group (n = 7814)	LTBI group (n = 1327)	Statistics	*P*	SMD	Control group (n = 3952)	LTBI group (n = 1327)	Statistics	*P*	SMD
Age (years)	31.05 ± 4.32	32.12 ± 4.63	t=-7.839	<.001^†^	0.231	32.05 ± 4.53	32.12 ± 4.63	t=-0.486	0.627	0.015
BMI (kg/m^2^)	21.93 ± 3.06	22.10 ± 3.01	t=-1.887	0.059	0.057	21.97 ± 3.02	22.10 ± 3.01	t=-1.289	0.198	0.041
bFSH (mIU/mL)	7.66 ± 2.58	7.90 ± 2.68	t=-3.128	0.002^†^	0.090	7.77 ± 2.61	7.90 ± 2.68	t=-1.646	0.100	0.051
AFC	12.00 (8.00, 19.00)	12.00 (8.00, 18.00)	Z=-3.315	<.001^†^	0.095	12.00 (8.00, 18.00)	12.00 (8.00, 18.00)	Z=-0.055	0.956	0.006
AMH (ng/mL)	3.59 (1.92, 6.40)	3.35 (1.69, 5.87)	Z=-3.388	<.001^†^	0.114	3.25 (1.71, 5.91)	3.35 (1.69, 5.87)	Z=-0.026	0.979	0.020
Duration of infertility (years)	3.00 (2.00, 4.00)	3.00 (2.00, 4.00)	Z=-0.969	0.333	0.073	3.00 (1.00, 4.00)	3.00 (2.00, 4.00)	Z=-1.929	0.054	0.079
Type of infertility, n (%)			χ²=2.357	0.125	0.045			χ²=2.673	0.102	0.053
Primary infertility	5423 (69.40)	893 (67.29)				2562 (64.83)	893 (67.29)			
Secondary infertility	2391 (30.60)	434 (32.71)				1390 (35.17)	434 (32.71)			
Cause of Infertility, n (%)										
Ovulation Dysfunction (except PCOS)	1117 (14.29)	203 (15.30)	χ²=0.923	0.337	0.028	625 (15.81)	203 (15.30)	χ²=0.201	0.654	0.014
PCOS	1550 (19.84)	233 (17.56)	χ²=3.748	0.053	0.060	697 (17.64)	233 (17.56)	χ²=0.004	0.948	0.002
Pelvic/Tubal Factor	3887 (49.74)	800 (60.29)	χ²=50.462	<.001^†^	0.215	2381 (60.25)	800 (60.29)	χ²=0.001	0.980	0.001
Endometriosis	641 (8.20)	86 (6.48)	χ²=4.597	0.032^†^	0.070	339 (8.58)	86 (6.48)	χ²=5.902	0.015^†^	0.085
Male Factor	2312 (29.59)	317 (23.89)	χ²=17.985	<.001^†^	0.134	935 (23.66)	317 (23.89)	χ²=0.029	0.865	0.005
Uterine Factor	1615 (20.67)	307 (23.13)	χ²=4.157	0.041^†^	0.058	945 (23.91)	307 (23.13)	χ²=0.332	0.565	0.018
Unexplained	713 (9.12)	83 (6.25)	χ²=11.753	<.001^†^	0.119	242 (6.12)	83 (6.25)	χ²=0.030	0.863	0.005
COH protocol, n (%)			χ²=8.991	0.029^†^				χ²=0.996	0.802	
GnRH agonist	4774 (61.10)	761 (57.35)			0.076	2293 (58.02)	761 (57.35)			0.014
GnRH antagonist	2588 (33.12)	474 (35.72)			0.054	1362 (34.46)	474 (35.72)			0.026
PPOS	387 (4.95)	83 (6.25)			0.054	269 (6.81)	83 (6.25)			0.023
Others	65 (0.83)	9 (0.68)			0.019	28 (0.71)	9 (0.68)			0.004
Fertilization Method, n (%)			χ²=13.101	<.001^†^	0.113			χ²=0.059	0.808	0.008
IVF	5502 (70.41)	999 (75.28)				2962 (74.95)	999 (75.28)			
ICSI	2312 (29.59)	328 (24.72)				990 (25.05)	328 (24.72)			

^*^ Values are presented as the mean±SD, median (25th percentile, 75th percentile) or proportion (%). ^†^ *P*<0.05 Abbreviations: PSM, propensity score matching; LTBI, latent tuberculosis infection; SMD, standardized mean difference; BMI, body mass index; bFSH, basal follicle stimulating hormone; AFC, antral follicle count; AMH, anti-Müllerian hormone; PCOS, polycystic ovary syndrome; COH, controlled ovarian hyperstimulation; GnRH, gonadotrophin release hormone; PPOS, progestin-primed ovarian stimulation; IVF, *in vitro* fertilization; ICSI, intracytoplasmic sperm injection.

**Table 5:** Comparisons of COH outcomes and cumulative pregnancy outcomes between the LTBI group and the control group^*^

	Control group (n=3952)	LTBI group (n=1327)	Statistics	*P*
Gn days (days)	10.04 ± 1.96	10.08 ± 1.90	t=-0.65	0.517
Gn dosage (IU)	2455.55 ± 866.27	2478.02 ± 836.70	t=-0.82	0.410
Number of >14 mm follicles on hCG day	10.40 ± 5.31	10.48 ± 5.34	t=-0.47	0.636
Number of oocytes retrieved	12.25 ± 7.14	12.27 ± 7.13	t=-0.10	0.922
Number of MⅡ oocytes	10.49 ± 6.25	10.57 ± 6.41	t=-0.41	0.684
Number of 2PN embryos	7.43 ± 4.84	7.46 ± 4.92	t=-0.18	0.856
Blastocyst formation rate, %(n)	69.18 (16853/24360)	70.53 (5857/8304)	χ²=5.32	0.201
Number of available embryos	4.11 ± 2.85	4.20 ± 3.03	t=-0.96	0.339
Conservative CPR, %(n)	70.42 (2783/3952)	70.16 (931/1327)	χ²=0.03	0.857
Optimistic CPR, %(n)	77.18 (3050/3952)	76.26 (1012/1327)	χ²=0.47	0.494
Conservative CLBR, %(n)	61.94 (2448/3952)	61.42 (815/1327)	χ²=0.12	0.733
Optimistic CLBR, %(n)	73.25 (2895/3952)	72.65 (964/1327)	χ²=0.19	0.665

^*^ Values are presented as the means±SDs or proportions (%). Abbreviations: COH, controlled ovarian hyperstimulation; LTBI, latent tuberculosis infection; Gn, gonadotrophin; IU, international unit; hCG, human chorionic gonadotrophin; MⅡ, metaphase II; 2PN, double pronucleus; CPR, cumulative pregnancy rate; CLBR, cumulative live birth rate.

**Table 6:** Multivariate logistic regression analysis of the related factors affecting CPR/CLBR

Variate	Conservative CPR		Optimistic CPR		Conservative CLBR		Optimistic CLBR	
	*P*	aOR (95%CI)	*P*	aOR (95%CI)	*P*	aOR (95%CI)	*P*	aOR (95%CI)
LTBI	0.841	0.98 (0.83 - 1.16)	0.500	0.93 (0.76 - 1.14)	0.460	0.95 (0.82 - 1.10)	0.552	0.94 (0.78 - 1.14)
Age	<.001^a^	0.91 (0.88 - 0.93)	<.001^*^	0.94 (0.91 - 0.97)	<.001^*^	0.91 (0.89 - 0.94)	<.001^*^	0.94 (0.91 - 0.97)
Number of oocytes retrieved	0.754	0.99 (0.95 - 1.04)	0.808	0.99 (0.94 - 1.05)	0.508	0.99 (0.95 - 1.02)	0.438	0.98 (0.93 - 1.03)
Number of available embryos	<.001^*^	1.53 (1.44 - 1.63)	<.001^*^	2.25 (2.04 - 2.48)	<.001^*^	1.34 (1.28 - 1.41)	<.001^*^	2.14 (1.97 - 2.34)
Number of MⅡ oocytes	0.830	0.99 (0.94 - 1.05)	0.740	1.01 (0.94 - 1.09)	0.623	1.01 (0.96 - 1.06)	0.213	1.04 (0.98 - 1.12)
Number of 2PN embryos	0.054	1.05 (1.00 - 1.10)	0.151	1.05 (0.98 - 1.12)	0.134	1.03 (0.99 - 1.08)	0.585	1.02 (0.96 - 1.08)
Endometriosis	0.492	0.90 (0.66 - 1.22)	0.176	0.77 (0.52 - 1.12)	0.674	0.94 (0.72 - 1.24)	0.758	0.95 (0.67 - 1.35)

^*^ *P*<0.05 Abbreviations: CPR, cumulative pregnancy rate; CLBR, cumulative live birth rate; aOR, adjusted odds ratio; LTBI, latent tuberculosis infection; MⅡ, metaphase II; 2PN, double pronucleus.

**Table 7:** Comparisons of pregnancy outcomes between the LTBI group and the control group undergoing fresh embryo transfer

	Control group (n=2545)	LTBI group (n=858)	Statistics	*P*
Biochemical pregnancy rate, %(n)	55.17 (1404/2545)	53.03 (455/858)	χ²=1.18	0.277
Clinical pregnancy rate, %(n)	48.17 (1226/2545)	46.15 (396/858)	χ²=1.05	0.306
Ectopic pregnancy rate, %(n)	0.79 (20/2545)	0.35 (3/858)	χ²=1.82	0.177
Live birth rate, %(n)	40.83 (1039/2545)	39.28 (337/858)	χ²=0.64	0.424
Multiple pregnancy rate, %(n)	5.87 (72/1226)	6.31 (25/396)	χ²=0.10	0.748
Miscarriage rate, %(n)	15.01 (184/1226)	14.65 (58/396)	χ²=0.03	0.861
Preterm birth rate, %(n)	8.24 (101/1226)	7.58 (30/396)	χ²=0.18	0.674
Maternal and neonatal complications rate, %(n)	20.88 (256/1226)	19.70 (78/396)	χ²=0.26	0.612

Abbreviations: LTBI, latent tuberculosis infection.

**Table 8:** Multivariate logistic regression analysis of the related factors affecting pregnancy outcomes in patients who underwent fresh embryo transfer

Variate	Clinical pregnancy		Live birth		Miscarriage	
	*P*	aOR (95%CI)	*P*	aOR (95%CI)	*P*	aOR (95%CI)
LTBI	0.405	0.93 (0.80 - 1.10)	0.694	0.97 (0.82 - 1.14)	0.525	0.90 (0.66 - 1.23)
Age	0.016 ^*^	0.96 (0.94 - 0.99)	0.003^*^	0.96 (0.93 - 0.98)	0.344	1.03 (0.97 - 1.09)
Number of MⅡ oocytes	0.068	0.97 (0.93 - 1.00)	0.177	0.98 (0.94 - 1.01)	0.175	0.95 (0.89 - 1.02)
Number of 2PN embryos	0.008^*^	1.06 (1.02 - 1.11)	0.019^*^	1.05 (1.01 - 1.10)	0.360	1.04 (0.96 - 1.13)
Endometrial thickness on hCG day	0.005 ^*^	1.05 (1.01 - 1.08)	0.040^*^	1.03 (1.01 - 1.07)	0.215	1.04 (0.98 - 1.11)
COH protocol						
GnRH agonist	Reference					
GnRH antagonist	<.001^*^	0.60 (0.50 - 0.72)	<.001^*^	0.64 (0.53 - 0.77)	0.086	0.72 (0.50 - 1.05)
Endometriosis	0.168	1.24 (0.91 - 1.69)	0.152	1.25 (0.92 - 1.71)	0.957	1.02 (0.59 - 1.75)
Number of transferred embryo(s)						
Single	Reference					
Double	0.047 ^*^	1.34 (1.01 - 1.79)	0.349	1.15 (0.86 - 1.53)	0.034^*^	1.74 (1.04 - 2.91)

^*^ *P*<0.05 Abbreviations: CPR, cumulative pregnancy rate; CLBR, cumulative live birth rate; aOR, adjusted odds ratio; LTBI, latent tuberculosis infection; MⅡ, metaphase II; 2PN, double pronucleus.

## Abbreviations

TB: Tuberculosis
MTB: *Mycobacterium tuberculosis*
HIV: human immunodeficiency virus
AIDS: acquired immune deficiency syndrome
LTBI: latent tuberculosis infection
IGRA: interferon-gamma release assay
OR: ovarian reserve
ART: assisted reproductive technology
IVF: *in vitro* fertilization
ICSI: intracytoplasmic sperm injection
PGT: preimplantation genetic testing
TESA: testicular sperm aspiration
PESA: percutaneous epididymal sperm aspiration
mTESE: microdissection testicular sperm extraction
COH: controlled ovarian hyperstimulation
GnRH: gonadotropin-releasing hormone
PPOS: progestin-primed ovarian stimulation
Gn: gonadotropin
BMI: body mass index
TVS: transvaginal ultrasonography
AMH: anti-Müllerian hormone
AFC: antral follicle count
bFSH: basal follicle-stimulating hormone
CLBR: cumulative live birth rate
CPR: cumulative pregnancy rate
PSM: propensity score matching
SMD: standardized mean difference
MII: metaphase II
2PN: double pronucleus
