# Supplementary material for: Impact of Latent Tuberculosis Infection on Ovarian Reserve and In Vitro Fertilization or Intracytoplasmic Sperm Injection Outcomes: A Retrospective Cohort Study with Propensity Score Matching
Source: Int J Med Sci. 2025 Oct 20;22(16):4384–95. doi: 10.7150/ijms.114851 (PMC12595342; doi:10.7150/ijms.114851)
Supplement: Supplementary file 1 — Supplementary tables. [file ijmsv22p4384s1.pdf]

Table S1: Baseline characteristics of non-PCOS women included in Analysis 1[a]

	Before PSM					After PSM				
	Control group (n = 7867)	LTBI group (n = 1261)	Statistics	*P*	SMD	Control group (n = 2515)	LTBI group (n = 1260)	Statistics	*P*	SMD
Age (years)	31.23±4.47	32.48±4.78	t=-8.634	<.001[b]	0.260	32.42±4.85	32.46±4.77	t=-0.241	0.810	0.008
BMI (kg/m^2)	21.71±2.95	22.02±2.98	t=-3.422	<.001[b]	0.103	21.99±3.02	22.01±2.98	t=-0.230	0.818	0.008
Duration of infertility (years)	2.50 (1.50, 4.00)	3.00 (2.00, 4.00)	Z=-1.827	0.068	0.107	3.00 (1.50, 4.00)	3.00 (2.00, 4.00)	Z=-0.300	0.764	0.030
ART method, n (%)			χ^2=38.009	<.001[b]	0.230			χ^2=0.067	0.795	0.009
Artificial Insemination	1271 (16.16)	119 (9.44)				231 (9.18)	119 (9.44)			
IVF/ICSI	6596 (83.84)	1142 (90.56)				2284 (90.82)	1141 (90.56)			
Type of infertility, n (%)			χ^2=8.950	0.003[b]	0.088			χ^2=0.004	0.948	0.002
Primary infertility	5621 (71.45)	849 (67.33)				1690 (67.20)	848 (67.30)			
Secondary infertility	2246 (28.55)	412 (32.67)				825 (32.80)	412 (32.70)			
Cause of Infertility, n (%)										
Ovulation Dysfunction (except PCOS)	1187 (15.09)	203 (16.10)	χ^2=0.859	0.354	0.027	434 (17.26)	203 (16.11)	χ^2=0.785	0.376	0.031
Pelvic/Tubal Factor	3431 (43.61)	693 (54.96)	χ^2=56.465	<.001[b]	0.228	1394 (55.43)	692 (54.92)	χ^2=0.087	0.768	0.010
Endometriosis	602 (7.65)	75 (5.95)	χ^2=4.599	0.032[b]	0.072	192 (7.63)	75 (5.95)	χ^2=3.613	0.057	0.071
Male Factor	2881 (36.62)	369 (29.26)	χ^2=25.669	<.001[b]	0.162	735 (29.22)	369 (29.29)	χ^2=0.002	0.969	0.001
Uterine Factor	1551 (19.72)	280 (22.20)	χ^2=4.200	0.040[b]	0.060	610 (24.25)	280 (22.22)	χ^2=1.924	0.165	0.049
Unexplained	1081 (13.74)	120 (9.52)	χ^2=16.976	<.001[b]	0.144	229 (9.11)	120 (9.52)	χ^2=0.175	0.676	0.014

[a] Values are presented as mean±SD, median (25th percentile, 75th percentile) or proportion (%).

[b] $P<0.05$

Abbreviations: PCOS, polycystic ovary syndrome; PSM, propensity score matching; LTBI, latent tuberculosis infection; SMD, standardized mean difference; BMI, body mass index; ART, assisted reproductive technology; IVF, *in vitro* fertilization; ICSI, intracytoplasmic sperm injection.

Table S2: Comparisons of bFSH, AMH and AFC between non-PCOS women with or without LTBI[a]

	Control group (n = 2515)	LTBI group (n = 1260)	Statistics	*P*
AMH (ng/mL)	3.78 ± 2.89	3.41 ± 2.59	t=4.55	<0.001[b]
bFSH (mIU/mL)	8.11 ± 3.25	8.18 ± 3.07	t=-0.72	0.472
AFC	11.39 ± 5.18	10.84 ± 5.20	t=3.53	<0.001[b]

[a] Values are presented as mean±SD.

[b] $P<0.05$

Abbreviations: bFSH, basal follicle stimulating hormone; AMH, anti-müllerian hormone; AFC, antral follicle count; PCOS, polycystic ovary syndrome; LTBI, latent tuberculosis infection.

Table S3: Baseline characteristics of women undergoing fresh embryo transfer in Analysis 2[a]

	Before PSM					After PSM				
	Control group (n = 5471)	LTBI group (n = 859)	Statistics	*P*	SMD	Control group (n = 2545)	LTBI group (n = 858)	Statistics	*P*	SMD
Age (years)	30.71 ± 3.87	31.61 ± 4.10	t=-6.026	<.001[b]	0.220	31.55 ± 4.02	31.59 ± 4.08	t=-0.288	0.773	0.011
BMI (kg/m^2)	22.00 ± 3.03	22.12 ± 3.03	t=-1.047	0.295	0.038	22.05 ± 2.96	22.12 ± 3.04	t=-0.603	0.547	0.023
bFSH (mIU/mL)	7.57 ± 2.28	7.84 ± 2.31	t=-3.146	0.002[b]	0.114	7.79 ± 2.41	7.83 ± 2.29	t=-0.347	0.728	0.014
AFC	12.00 (9.00, 18.00)	11.00 (8.00, 17.00)	Z=-3.545	<.001[b]	0.119	12.00 (8.00, 17.00)	11.00 (8.00, 17.00)	Z=-0.569	0.569	0.019
AMH (ng/mL)	3.52 (2.01, 6.06)	3.16 (1.73, 5.47)	Z=-3.537	<.001[b]	0.147	3.12 (1.81, 5.43)	3.16 (1.73, 5.48)	Z=-0.145	0.885	0.012
Duration of infertility (years)	3.00 (2.00, 4.00)	3.00 (2.00, 4.00)	Z=-0.141	0.888	0.053	3.00 (1.50, 4.00)	3.00 (2.00, 4.00)	Z=-0.579	0.562	0.042
Type of infertility, n (%)			χ^2=3.181	0.075	0.064			χ^2=0.071	0.790	0.011
Primary infertility	3827 (69.95)	575 (66.94)				1690 (66.40)	574 (66.90)			
Secondary infertility	1644 (30.05)	284 (33.06)				855 (33.60)	284 (33.10)			
Cause of Infertility, n (%)										
Ovulation Dysfunction (except PCOS)	664 (12.14)	119 (13.85)	χ^2=2.018	0.155	0.050	353 (13.87)	118 (13.75)	χ^2=0.007	0.931	0.003
PCOS	965 (17.64)	132 (15.37)	χ^2=2.674	0.102	0.063	398 (15.64)	132 (15.38)	χ^2=0.031	0.859	0.007
Pelvic/Tubal Factor	2772 (50.67)	516 (60.07)	χ^2=26.295	<.001[b]	0.192	1495 (58.74)	516 (60.14)	χ^2=0.518	0.472	0.029
Endometriosis	427 (7.80)	51 (5.94)	χ^2=3.710	0.054	0.079	216 (8.49)	51 (5.94)	χ^2=5.740	0.017[b]	0.108
Male Factor	1652 (30.20)	208 (24.21)	χ^2=12.801	<.001[b]	0.140	619 (24.32)	208 (24.24)	χ^2=0.002	0.962	0.002
Uterine Factor	1052 (19.23)	179 (20.84)	χ^2=1.228	0.268	0.040	563 (22.12)	178 (20.75)	χ^2=0.713	0.398	0.034
Unexplained	562 (10.27)	60 (6.98)	χ^2=9.056	0.003[b]	0.129	182 (7.15)	60 (6.99)	χ^2=0.024	0.876	0.006
COH protocol, n (%)			χ^2=4.834	0.089				χ^2=0.369	0.832	
GnRH agonist	3554 (64.96)	527 (61.35)			0.074	1575 (61.89)	527 (61.42)			0.010

	Before PSM					After PSM				
	Control group (n = 5471)	LTBI group (n = 859)	Statistics	*P*	SMD	Control group (n = 2545)	LTBI group (n = 858)	Statistics	*P*	SMD
GnRH antagonist	1897 (34.67)	330 (38.42)			0.077	961 (37.76)	329 (38.34)			0.012
Others	20 (0.37)	2 (0.23)			0.028	9 (0.35)	2 (0.23)			0.025
Fertilization Method, n (%)			χ^2=5.105	0.024[b]	0.086			χ^2=0.174	0.676	0.017
IVF	3851 (70.39)	637 (74.16)				1868 (73.40)	636 (74.13)			
ICSI	1620 (29.61)	222 (25.84)				677 (26.60)	222 (25.87)			

[a] Values are presented as mean±SD, median (25th percentile, 75th percentile) or proportion (%).

[b] $P<0.05$

Abbreviations: PSM, propensity score matching; LTBI, latent tuberculosis infection; SMD, standardized mean difference; BMI, body mass index; bFSH, basal follicle stimulating hormone; AFC, antral follicle count; AMH, anti-müllerian hormone; PCOS, polycystic ovary syndrome; COH, controlled ovarian hyperstimulation; GnRH, gonadotrophin release hormone; IVF, *in vitro* fertilization; ICSI, intracytoplasmic sperm injection.

Table S4: Comparisons of COH outcomes between LTBI group and control group undergoing fresh embryo transfer[a]

	Control group (n=2545)	LTBI group (n=858)	Statistics	*P*
Gn days (days)	10.05 ± 1.85	10.12 ± 1.92	t=-0.94	0.345
Gn dosage (IU)	2492.24 ± 864.71	2527.31 ± 832.87	t=-1.04	0.300
Number of >14mm follicles on hCG day	9.81 ± 4.31	9.78 ± 4.40	t=0.16	0.873
Endometrial thickness on hCG day (mm)	11.58 ± 2.54	11.48 ± 2.62	t=1.03	0.304
Number of oocytes retrieved	11.27 ± 5.22	10.92 ± 5.20	t=1.73	0.084
Number of MII oocytes	9.64 ± 4.65	9.36 ± 4.75	t=1.51	0.131
Number of 2PN embryos	6.81 ± 3.79	6.66 ± 3.74	t=1.04	0.298
Number of transferred embryo(s), %(n)			χ^2=0.55	0.457
Single	90.61 (2306/2545)	89.74 (770/858)		
Double	9.39 (239/2545)	10.26 (88/858)		

[a] Values are presented as mean±SD or proportion (%).

Abbreviations: COH, controlled ovarian hyperstimulation; LTBI, latent tuberculosis infection; Gn, gonadotrophin; IU, international unit; hCG, human chorionic gonadotrophin; MII, metaphase II; 2PN, double pronucleus.
